# Supplementary material for: Chromothripsis during telomere crisis is independent of NHEJ, and consistent with a replicative origin
Source: Genome Res. 2019 May;29(5):737–49. doi: 10.1101/gr.240705.118 (PMC6499312; doi:10.1101/gr.240705.118)
Supplement: Supplemental Material [file supp_gr.240705.118_Supplemental_file_1.zip › contigs/annotated_contigs/DB96/contig.2.DB96_length_528_mean_cov_8.35606060606.docx]

**DB96_length_528_mean_cov_8.35606060606**

```
TTAAAAAAAGGGCTGGGCATGGTGGCCCACATCCGTAATCCCAGCACTTTGGGAGGCCAAGGCAGGCAGATCACTTGAGGTCAGGAGTT
 >chr6:151511509-151511833 + E=2e-183
CGAGGTCAGCCTGGCCAACATGGTGAAATCTCTACTAAAAATACAGAAATTAGCCAGACGTGGTGGACCGTATCTGTACTCCCAGCTAC

TTGGGAGGCTGAGGCAGGAGAATCACTTGAACCCAGGAGGCAGAGGTTGCAGTGAGCCAAGATTGCGCCACTGCACTTCAGCCTGGGTG

ACAGACTGAGACTCCGTCTCAAAAGAGCAATTGAGCCCGGGTGCGGTGGCTCACGCC|ACA|TCAGGTGGGAAGCTCTGGCTTAGGAAA
                                                             >chr6:151358704-151358905
TTACTGAATCATAGGAGTTCTCATTTTTGATGTTGTTGTGATGGGATCATGTTGCTATTGGATGATTATTCTGTTATTTTGTGTTATCT
+ E=2e-109
ATGGGGGTAAAGGAGATGGGCAGATAAATAAAAGCTTATCAAAGTGCTTAAAATCCATGAAGATGGGCTTCATTTGGTACAGCTA
```
